# Stercoral colitis in the emergency department: a review of the literature

**DOI:** 10.1186/s12245-023-00578-x

**Published:** 2024-01-02

**Authors:** Emily Bae, Jacqueline Tran, Kaushal Shah

**Affiliations:** 1 Weill Cornell Medicine, Weill Cornell Medical College, 1300 York Ave, New York, NY 10065 USA; 2 Weill Cornell Medicine, Emergency Medicine, New York Presbyterian Hospital, 525 East 68Th Street, New York, NY 10065 USA

**Keywords:** Stercoral colitis, Constipation, Fecal impaction, CT imaging, Stercoral ulceration, Perforation

## Abstract

**Background:**

Stercoral colitis (SC) is a rare but potentially life-threatening inflammatory colitis caused by the accumulation of impacted fecal material. Despite reported associations with bowel perforation and high mortality rates, stercoral colitis remains a poorly defined and underrecognized diagnosis in the emergency department (ED).

**Objective of the review:**

This review aims to summarize and synthesize existing literature on SC to guide its recognition and management in the ED.

**Discussion:**

SC primarily occurs in elderly or bedbound patients with chronic constipation; however, it does occur in younger patients with comorbidities at increased risk for fecal impaction. Patients may present acutely with abdominal pain and distension, but clinical presentation is often nonspecific and varied, and there are no established diagnostic criteria for SC to date. CT is therefore crucial for diagnosis, revealing key findings such as fecaloma, colonic dilatation, and fat stranding. Treatment depends on severity of illness, ranging from manual disimpaction and other conservative measures for most cases, to surgical intervention for complicated cases, such as stercoral perforation.

**Conclusions:**

SC can be a challenging diagnosis in the ED, often requiring multidisciplinary collaboration. Timely recognition and appropriate treatment are essential to reduce morbidity and mortality associated with this condition. Further research is needed to establish diagnostic criteria and clear management algorithms.

## Introduction

Stercoral colitis (SC) is an uncommon but potentially life-threatening condition that can present acutely in the emergency department (ED). Typically the sequela of chronic constipation, it is characterized by impacted fecal material causing increased colonic intraluminal pressure and inflammation. If not recognized and treated in a timely manner, stercoral colitis may lead to dangerous complications such as ischemic necrosis, perforation, and/or sepsis, with a mortality rate up to 63% when severe complications arise [1, 2].

In the ED, SC is typically a radiologic diagnosis. While it should be considered in any patient with chronic constipation and abdominal pain, clinical diagnosis is made challenging by the fact that patients often present with vague signs and symptoms, or they may be altogether asymptomatic [1–4]; in fact, recent literature has reported that 60% of SC patients in the ED denied any abdominal pain [5]. Computed Tomography (CT) imaging and clinical intuition are therefore critical to the diagnosis of SC, and several studies have described radiologic findings important for the detection of SC and its complications.

Although there are guidelines for the management of chronic constipation, there are no established evidence-based guidelines for the diagnosis and management of SC [6]. Management is variable depending on the clinical scenario and severity of the patient’s condition. Since the clinical picture can rapidly deteriorate into a surgical emergency or sepsis, it is critical for ED physicians to maintain a high index of suspicion for SC in patients who may have a history of constipation, particularly in the elderly population.

Since the first report in 1894, SC has mainly been described in surgical and gastrointestinal literature in the form of case reports [7, 8]. Only a handful of studies have investigated SC as it pertains to the emergency medicine setting. Given the paucity of knowledge on the topic, this review aims to summarize the current understanding of SC, including its epidemiology, pathogenesis, clinical presentation, diagnosis, and management in the ED.

## Discussion

### Pathophysiology

SC is an inflammatory colitis that typically occurs in the setting of chronic constipation when impacted fecal material causes increased intraluminal pressure in the colon. Its pathogenesis was first postulated by Serpell and Nicholls in 1990, who reviewed 64 reported cases of stercoral perforation and observed that the disease involves an entire segment of colon rather than a single focal point of perforation. Furthermore, they recognized that fecal impaction was necessary to initiate the development of ischemic pressure necrosis [9, 10].

The name “stercoral” comes from the Latin word *stercus* for feces. In SC, hard, dry fecal matter accumulates and becomes lodged within the hypomobile bowel [11]. This mass of dehydrated stool, termed a fecaloma or stercoroma, exerts pressure on the intestinal walls, resulting in marked colonic distension [3, 7, 9, 10]. The vasculature within the surrounding bowel wall becomes compressed, leading to edema and inflammation. Eventually, the increased intraluminal pressure exceeds capillary perfusion pressure in the bowel wall, compromising regional vascular supply and transmural perfusion [3, 9, 12, 13]. As blood supply fails to meet metabolic demands, a variety of feared ischemic complications may result, including pressure necrosis, ulceration, and ultimately perforation. If perforation occurs, fecal contamination of the peritoneal cavity can lead to sepsis and death.

The three most common anatomic locations affected by SC are the apex of the sigmoid colon, the antimesenteric border of the rectosigmoid junction, and the anterior rectum [1, 7, 12, 14, 15]. These regions are thought to be more susceptible due to several factors that contribute to significantly increased intraluminal pressure: (1) decreasing water content of the stool as water gets absorbed along the colon, (2) relatively narrow luminal diameter, particularly at the rectosigmoid junction, makes it hard for stool to pass through, and (3) poor perfusion to these regions, especially over the antimesenteric aspect of the bowel, because blood enters from the mesenteric side [1, 12, 16]. Most reported cases of stercoral ulceration have been described as occurring on the antimesenteric aspect of the bowel wall [7, 17, 18]. The rectosigmoid junction is most prone to stercoral ischemia and perforation, as this junction contains Sudeck’s point—a watershed region in the arterial supply containing minimal collateral circulation between branches of the inferior mesenteric and superior rectal arteries [12, 19].

### Epidemiology

SC most commonly affects elderly patients with chronic constipation, particularly those who are bedridden, residing in a nursing home, or with neurocognitive impairment, as they are at increased risk for fecal impaction [2, 7, 20]. The mean age of onset is thought to be over 60 [21]; however, cases have been described in younger patients with chronic opioid use, psychiatric conditions, and comorbidities predisposing to prolonged constipation such as hypothyroidism, diabetes, and renal failure [12, 20, 22]. Rare cases are seen in pediatric patients with a history of constipation [11]. A recent study of 269 cases of SC in the ED reported a median age of 76, though there was a range between six and 98 years (5). In general, patients with SC typically have some intrinsic or extrinsic etiologies for bowel hypomobility. Intrinsic factors may include functional, metabolic, or neurologic disorders, whereas extrinsic factors typically relate to pharmacologic side effects, as in the use of narcotics, NSAIDS, antacids, anticholinergics, antidepressants, etc. [14, 23–25]. Prolonged constipation, of course, is the single most important risk factor [24].

The incidence of SC is not well established for several reasons. In general, it is a rare condition and existing studies have been small-scale. The number of cases is thought to be historically underreported due to under-recognition, underdiagnosis, and misdiagnosis, as presenting symptoms may be vague and resemble other conditions such as diverticulitis. This is compounded by the fact that diagnosis of SC typically requires imaging studies, which may not be obtained in patients with non-specific symptoms. And even if imaging is obtained, there are currently no standardized criteria for diagnosis of SC.

While the true incidence and prevalence of SC and its complications are not known (1), the relative frequency of stercoral perforation has been described in a handful of surgical and pathological studies, mostly from the twentieth century. An autopsy study found the post-mortem incidence of stercoral perforation to range from 0.04 to 2.3%, though these statistics are thought to be underestimated [1, 17, 26]. Another study identified 1295 cases from a surgical database of patients with colorectal disease who underwent laparotomy intervention between 1993 and 1998. Stercoral perforation was present in 0.5% (*n* = 7 patients) of all surgical laparotomy procedures they studied, 1.2% of those surgeries that were emergent, and 3.2% of all colonic perforations identified during surgery. The authors then reviewed all published cases of stercoral perforation since its first report in 1984 to 1998; of 81 cases, the median age was 62 years (range 20–86), and 58% of patients were female [17]. More recently, a systematic review of stercoral perforation incorporating articles between 1998 and 2011 identified 137 patients with stercoral perforation. The median age was again 62 years (range 4–106) years, suggesting that the risk of perforation is not preferential to the elderly. Interestingly, stercoral perforation occurred more frequently in females by 30% [27]. As the majority of patients do not progress to perforation, these statistics represent a small subset of SC cases [21].

Despite the lack of epidemiological data, the incidence of SC is thought to be rising as emergency practitioners are ordering more imaging tests, average life expectancy gets longer, and the number of patients living with comorbidities and/or immobility increases [5, 28]. Chronic constipation is present in one-third of adults over age 60 worldwide, and large-scale epidemiological studies show that the high prevalence of chronic constipation is associated with age progression [29, 30]. Furthermore, up to 80% of institutionalized patients are affected by constipation [21]. A cross-sectional study of 34 randomly selected nursing homes in Spain found that 70% of residents had chronic constipation and 47% had experienced fecal impaction [31].

In addition to the aging population, chronic opioid use is an emerging risk factor for chronic constipation and SC [5]. As a growing proportion of the population is at risk for SC, which has the potential for high morbidity and mortality, it is critical for providers to be aware of this diagnosis as a distinct clinical entity.

### Clinical presentation and diagnosis

#### Signs and symptoms

The clinical presentation of SC is nonspecific and variable, thus the astute clinician must maintain a high index of suspicion to make the diagnosis. The classic presentation of SC is an elderly patient with multiple comorbidities, limited mobility, and a history of chronic constipation presenting with vague abdominal pain and distension accompanied by nausea and vomiting [3, 32, 33]. SC differs from other colitis clinically by the absence of diarrhea [26]. However, more than half of patients with a final diagnosis of SC will have an atypical presentation. In a 2023 study, Keim and colleagues reviewed initial ED visits for 269 patients with suspected or confirmed SC identified on CT across 21 hospitals; the most common chief complaints in their cohort included abdominal pain/distention (33.8%), constipation (17.8%), and nausea/vomiting or diarrhea (12.6%). Strikingly, this study revealed that abdominal pain was documented as absent in over 60% of patients, and over 25% of patients lacked abdominal tenderness on physical examination [5]. While most case reports describe patients with abdominal pain and tenderness, few have similarly reported cases where the abdominal examination was initially benign in the ED [22]. Fever, leukocytosis, and elevated acute phase reactants have also been associated with SC, though Keim’s group noted that the majority of patients in their cohort lacked any of those findings [5, 33, 34].

In rare cases, SC can progress to fatal complications such as ischemic necrosis, ulceration and perforation of the bowel, peritonitis, and septic shock. Patients with complicated SC may present with abdominal pain and signs of sepsis such as fever, hemodynamic instability, and leukocytosis [12, 32]. Case reports suggest that abdominal pain in SC complicated by ischemic colitis tends to be colicky in nature, while that in stercoral perforation is sudden and severe [1, 18, 35]. Physical examination typically reveals abdominal distension, tenderness to palpation, and—in the case of perforation—frank peritoneal signs. Unlike in classic bowel obstruction, patients typically have stool present in the rectal vault on the digital exam and may continue to pass stool and gas. It is important to note that, like in uncomplicated SC, clinical presentation can range from asymptomatic to frank peritoneal signs. In a review of 10 patients with surgically and pathologically confirmed necrotic SC, all patients presented to the ED with acute abdomen, but only 2 out of 10 patients exhibited clinically evident peritonitis [26].

There is no pathognomonic symptom or constellation of symptoms specific to SC or its complications. Nonspecific presentation makes it difficult to differentiate from constipation [12]. Diagnosis can be further delayed by comorbidities with overlapping signs and symptoms or concomitant neurologic and psychiatric disorders that mask physical examination findings. SC and its sequelae can mimic a range of other acute diagnoses, including diverticulitis, appendicitis, bowel obstruction, colon cancer perforation, mesenteric ischemia, and infectious colitis [5, 14, 16, 36, 37]. Clinicians who suspect a diagnosis of SC should pursue imaging to confirm the diagnosis and guide management.

#### Imaging findings

SC can be confirmed surgically and histopathologically, but in the acute setting, the diagnosis is primarily radiologic [17]. CT of the abdomen and pelvis with contrast is the gold standard for diagnosing SC and stercoral perforation [5, 36, 38]. CT accuracy for diagnosing stercoral perforation has been estimated at 82–90% [14, 15]. Plain radiographs may suggest the diagnosis, but they are relatively nonspecific and unreliable. Abdominal radiographs can indicate fecal impaction and upright chest radiographs can be helpful for patients in whom perforation or peritonitis is suspected; however, chest radiographs show free air under the diaphragm in only 30% of colonic perforations. One case study of stercoral perforation noted that pneumoperitoneum was readily identified on CT but consistently absent on plain radiography [12].

**Fig. 1:**
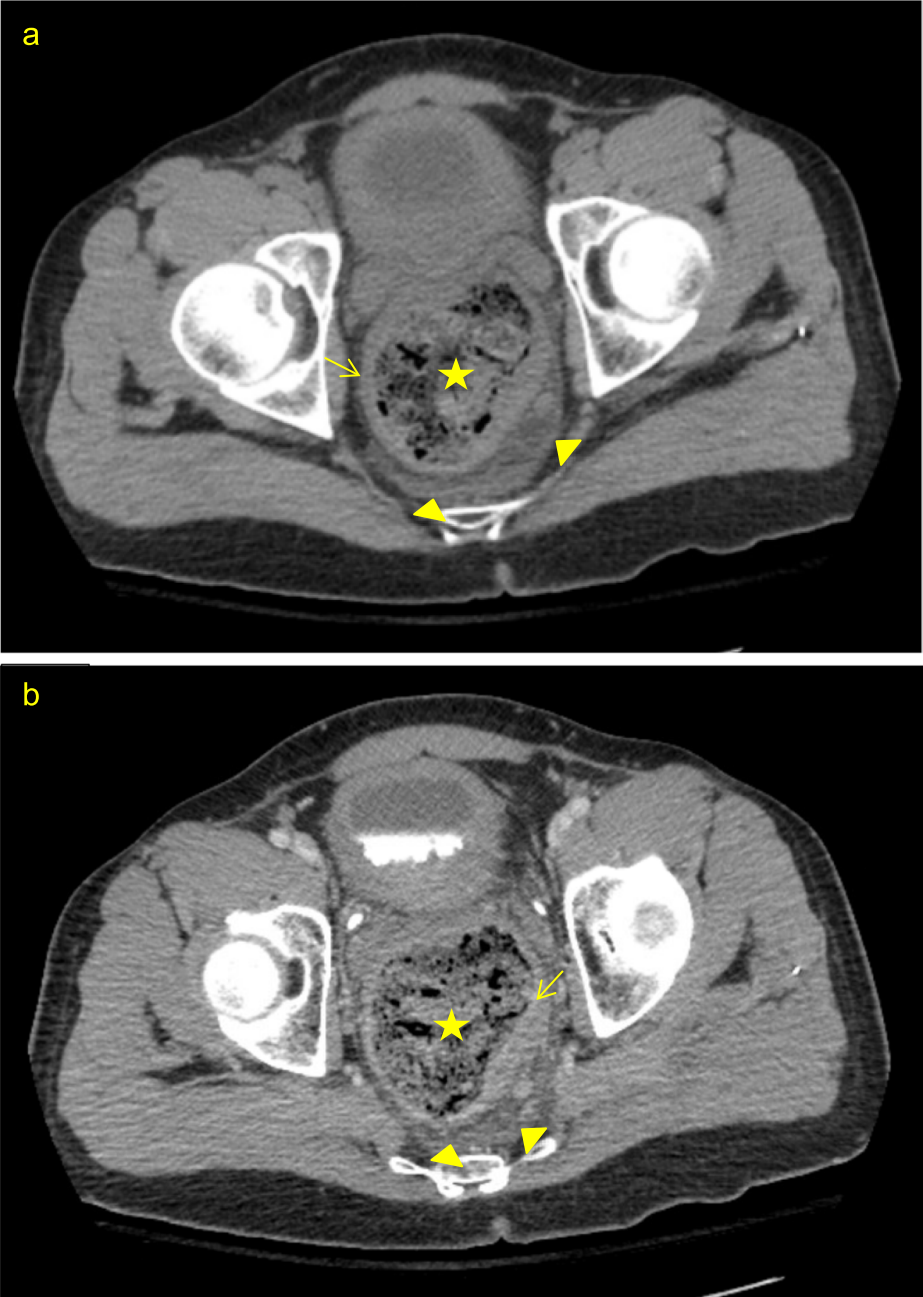
Axial pre-contrast (**a**) and contrast-enhanced (**b**) CT images of a 30-year-old male with stercoral colitis demonstrate rectal dilatation (star) due to fecal impaction. Rectal wall thickening (arrow) and perirectal fat stranding (arrowheads) are also seen as a result of inflammation

**Fig. 2:**
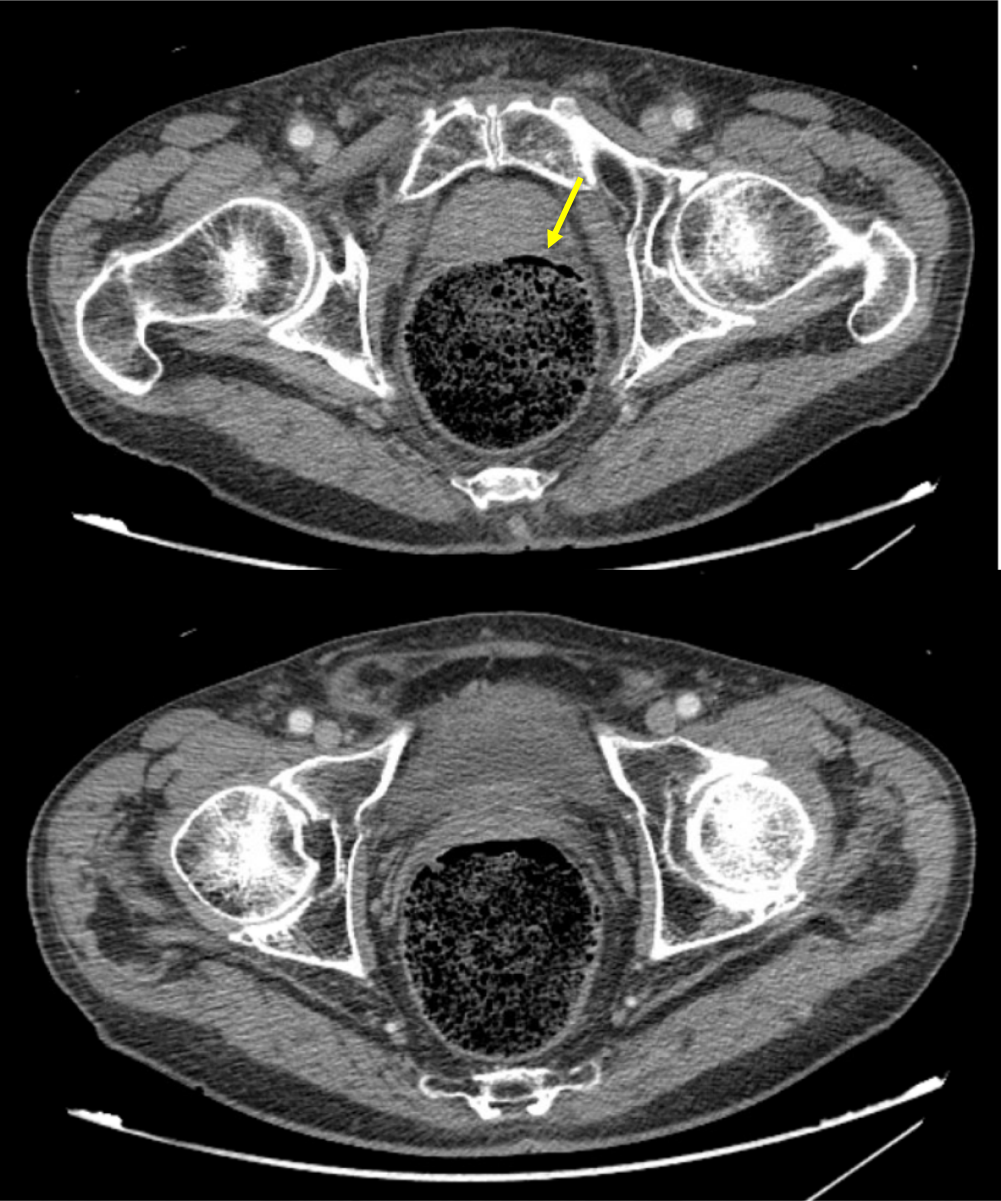
Axial contrast-enhanced CT images of an 89-year-old male with large colonic stool burden, mild rectal wall thickening, and fat stranding, suggesting stercoral colitis. Extraluminal air is seen within the rectal wall or rectal prostatic space, concerning for stercoral ulceration (arrow)

A number of studies have delineated key findings on CT that suggest the presence of SC, though no specific criteria have been proposed or validated in the current literature. Table 1 lists several radiologic features of SC. Presence of a fecaloma, colonic dilatation, thickening of the colon wall, and fat stranding, as seen in Fig. 1, are most commonly associated with SC. The presence of free air or abscess typically indicates that stercoral perforation has occurred [7]. Figure 2 depicts a case of potential stercoral ulceration, as evidenced by foci of extraluminal air near the rectal wall. Keim and colleagues reported that, across 269 SC patients in the ED, the most common CT findings accompanying the diagnosis included large stool burden/fecal impaction/fecaloma/inspissated stool (96.7%), bowel wall thickening/inflammation/mucosal hyperenhancement/fat stranding (48.3%), free fluid (9.7%), mesenteric edema (9.7%), and pneumatosis/bowel wall gas (7.4%) [5].

**Table 1:** Key CT findings in stercoral colitis

CT findings
Fecal impaction or fecaloma [3, 7, 33, 38]
Colonic dilatation [3, 7, 33, 38]
Pericolic fat stranding [3, 7, 33, 38]
Wall thickening of affected colon segment > 3 mm [7, 33, 38]
Extraluminal free air [3, 33, 38]
Pericolic abscess [7, 33, 38]
Mucosal discontinuity [33, 38]
Free fluid [33, 38]
Dense mucosa (pre-contrast CT) [38]

Some CT findings have been associated with poor outcomes in SC. Upon review of CT findings for 41 patients, Unal and colleagues found that increased length of the affected colon (> 40 cm) was significantly associated with mortality in SC (*P* = 0.010) [33]. Another study looked at the value of CT for discriminating fatal from nonfatal cases of SC; CT signs of dense mucosa, perfusion defects, ascites, and the presence of abnormal gas correlated with fatal SC [38]. Dense mucosa and perfusion defects, along with pericolonic stranding, were also found to be the most sensitive signs for detecting necrotic SC in another study by Wu and colleagues; however, this study was not powered to determine statistical significance for these CT features [26].

#### Pathologic findings

Stercoral ulceration and necrosis can be confirmed by surgical and histological findings [17]. Stercoral ulcers are usually found on the antimesenteric side of the colon, 1–10 cm in size with sharp margins, and occasionally multiple [26]. Other intraoperative findings include generalized peritonitis, colonic dilatation, and adjacent bowel wall edema. Histology of tissue from the perforated site has been found to show transmural necrosis, ulcer margins with sharp demarcation without undermining, nonspecific inflammatory changes with mononuclear cells in the lamina propria, and crypt abscesses [7, 26].

Maurer et al. are often cited for proposing four diagnostic criteria for colonic perforation: (1) a round and ovoid antimesenteric colonic perforation larger than 1 cm in diameter (2) the colon is full of stool that protrudes through the perforation site (3) microscopic evidence of multiple pressure ulcer and acute inflammatory reactions surrounding the perforation (4) absence of external injury, diverticulitis, or obstruction due to neoplasm or adhesions [17]. However, these criteria, published in 2000, are based on laparotomy findings and are of limited use for pre-operative recognition of SC. Most cases of uncomplicated SC are treated conservatively, i.e., with disimpaction, so histopathology is rarely obtained except in the case of surgical intervention [7].

### Management

While there are no official clinical practice guidelines for the management of SC, general principles for treatment have been proposed in the literature. The primary goal of treatment is to relieve pressure inside the colon by removal of the obstructing fecaloma, thereby addressing the underlying culprit: constipation.

#### Medical management

Appropriate management depends on the individual clinical scenario (e.g., severity of disease and patient comorbidities). In patients with uncomplicated SC, without any signs of peritonitis, nonoperative therapy is indicated. Conservative treatment tools include oral bowel regimen, enema, fecal disimpaction, and admission for close monitoring [2, 29, 32]. Pain control with opioids should be avoided to prevent worsening constipation [29]. Disimpaction can be done manually, medication-facilitated, or with endoscopic guidance; some publications support endoscopic disimpaction as the standard of care for nonoperative management of SC [1, 39]. Given that SC can rapidly progress to perforation or sepsis, emergency clinicians should consider surgical consults and keep patients NPO even in seemingly uncomplicated cases. It would be prudent to monitor blood cultures and sensitivity profiles, and if a patient develops fever, leukocytosis, or elevated lactate, antibiotics should be strongly considered.

#### Surgical management

If there﻿ is any concern for perforation, patients require immediate surgical evaluation and aggressive treatment with intravenous fluids and broad-spectrum antibiotics (covering gram-negative and anaerobic organisms), as sepsis-associated mortality risk increases substantially [39]. Operative intervention is a last resort reserved for patients with perforated SC or failure of conservative management [2].

According to existing literature, aggressive treatment involving resection of the entire affected bowel and the creation of a colostomy (i.e., Hartmann procedure) results in the best outcomes [17, 18]. Primary repair and more limited surgeries have traditionally been approached with caution, as they may be associated with greater mortality due to the risk of recurrent perforations in any affected colonic segments left behind [17]. In a review of 64 reported cases of stercoral perforation, Serpell and Nicholls stratified cases by surgical extent and found that postoperative mortality was lowest following resection of the entire diseased colon and colostomy (32%), compared to exteriorization of the colon without resection (43%) and closure of the perforation with proximal colostomy (57%) [9].

#### Multidisciplinary collaboration is critical

Management of SC requires a multidisciplinary effort. For the ED physician, timely care coordination with medicine, gastroenterology, and surgery is essential for optimal outcomes. Surgical consults should be placed early in the clinical course. Prompt recognition and treatment of SC are critical to prevent the high mortality associated with perforation and peritonitis [1].

### Complications and mortality

Sequelae of SC include perforation, ischemic necrosis, sepsis, and septic shock. Although rare, these complications are potentially fatal and require expedient diagnosis and management.

Stercoral perforation is an important complication of SC, with a mortality rate estimated at around 35% [9, 27]. Higher mortality has been reported in the literature, and rates seem to depend on management. In a 1990 review article, Serpell and Nicholls reported 32–57% post-operative mortality for stercoral perforation depending on the surgical technique used [9]. In 1998, Kanwal et al. reported the mortality rate of perforated SC to be 32% when surgically treated, and as high as 47% when managed conservatively with bowel regimen and antibiotics [14]. A more recent 2013 systematic review of stercoral perforation involving 137 patients estimated the overall mortality to be 34% [27]. Furthermore, mortality due to stercoral perforation may be underestimated, as in cases of sudden death of an elderly patient or bowel perforation attributed to another cause.

Patients with SC can have sepsis and septic shock with or without perforation. In one study looking at mortality in 11 cases of non-perforated SC with sepsis compared to mortality in 23 cases of perforated stercoral colitis, non-perforated SC with sepsis had a higher mortality rate of 64% compared to 35% in perforated SC. Their findings suggest that sepsis and lack of source control may be a bigger determinant of mortality than perforation status [2]. Given that sepsis alone carries a high mortality rate, these findings are not surprising. While this study was not statistically powered to conclude sepsis is a bigger predictor of mortality than perforation, it poses an important question regarding what factors should prompt surgical versus conservative management. The individual roles of sepsis and perforation in predicting mortality are areas for future exploration.

### Disposition

Interestingly, Keim et al. reported that of 269 patients in the ED with SC, 68.8% of patients were admitted, and of the remaining 84 patients that were discharged, half received no recommended treatment for SC [5]. This data highlights the reality that SC is a relatively unfamiliar diagnosis that is not only underrecognized but undertreated in practice. While current literature advocates for admission and close monitoring of patients with SC, it is possible that patients with uncomplicated cases may be safely treated at home. However, in the absence of data and clear management algorithms, this clinical decision-making is nebulous and remains in the clinical judgment of the clinician. Keim and colleagues astutely point out that, other than signs of perforation, the lack of data on other high-risk characteristics that suggest a need for admission remains a large gap in the literature [5].

## Conclusion

This review summarizes what is known regarding SC, which despite its associations with fatal complications, remains a poorly defined and understudied clinical entity. SC primarily affects elderly individuals with a history of constipation but should be considered in all patients at risk for chronic constipation and fecal impaction. Clinical presentation is often nonspecific and varied, and emergency physicians should pursue CT imaging when the diagnosis is suspected. While nonoperative and surgical treatments have been described, which patients require antibiotics, fluids, or other supportive measures remains unknown. More investigation is necessary to establish guidelines for diagnosis, management, and disposition.

## Data Availability

Not applicable.

## Abbreviations

SC: Stercoral colitis
ED: Emergency Department
CT: Computed tomography
